# Malignant reninoma with pulmonary metastasis and ATRX mutation treated with olaparib: a case report of specialized treatment

**DOI:** 10.3389/fimmu.2026.1803771

**Published:** 2026-05-04

**Authors:** Guo run Zi, Da-jiang Zhang, Dong-Lin He, Run-lin Feng, Chang-xing Ke

**Affiliations:** 1 Department of Urology, The Second Affiliated Hospital of Kunming Medical University, Kunming, China; 2 Department of Pathology, The Second Affiliated Hospital of Kunming Medical University, Kunming, China

**Keywords:** genitourinary cancer, immune checkpoint inhibitor, kidney cancer, next generation sequencing - NGS, relapse

## Abstract

We report a rare case of 32-year-old female with malignant reninoma complicated by pulmonary metastasis and ATRX mutation (p.G1964*, VAF 23.73%), initially misdiagnosed as renal pelvic carcinoma and treated with radical left nephrectomy and ureterectomy. The patient developed local recurrence and pancreatic head metastasis in January 2025, with stable disease achieved after radiotherapy, EP chemotherapy and toripalimab immunotherapy. Subsequent olaparib targeted therapy led to resolution of renal recurrent tumor, but progressive pancreatic lesion and persistent pulmonary metastasis were observed, suggesting tumor heterogeneity. This case demonstrates the value of NGS in screening actionable molecular targets for rare malignant reninoma; olaparib yields favorable local control for ATRX-mutated malignant reninoma based on synthetic lethality, while the differential response at metastatic sites underscores the need for exploring combination therapies to overcome tumor heterogeneity and drug resistance, providing a clinical reference for precision treatment of this rare disease.

## 1 Introduction

Juxtaglomerular cell tumor (JGCT), or reninoma, is an exceptionally rare renal neoplasm arising from the specialized cells of the juxtaglomerular apparatus (1). Since its initial description in 1967, only about 100 to 200 cases have been documented globally (2–4). While typically benign and curable through surgery, malignant variants with distant metastases are vanishingly rare and lack standardized management protocols. The classic clinical presentation includes hypertension, hypokalemia, and elevated plasma renin activity (5, 6). Recent research has identified NOTCH1 gene rearrangements and increased NOTCH signaling as key drivers in many cases (7). We present a unique case of malignant reninoma characterized by an ATRX mutation and its response to the PARP inhibitor olaparib.

## 2 Case presentation

**Figure 1:**
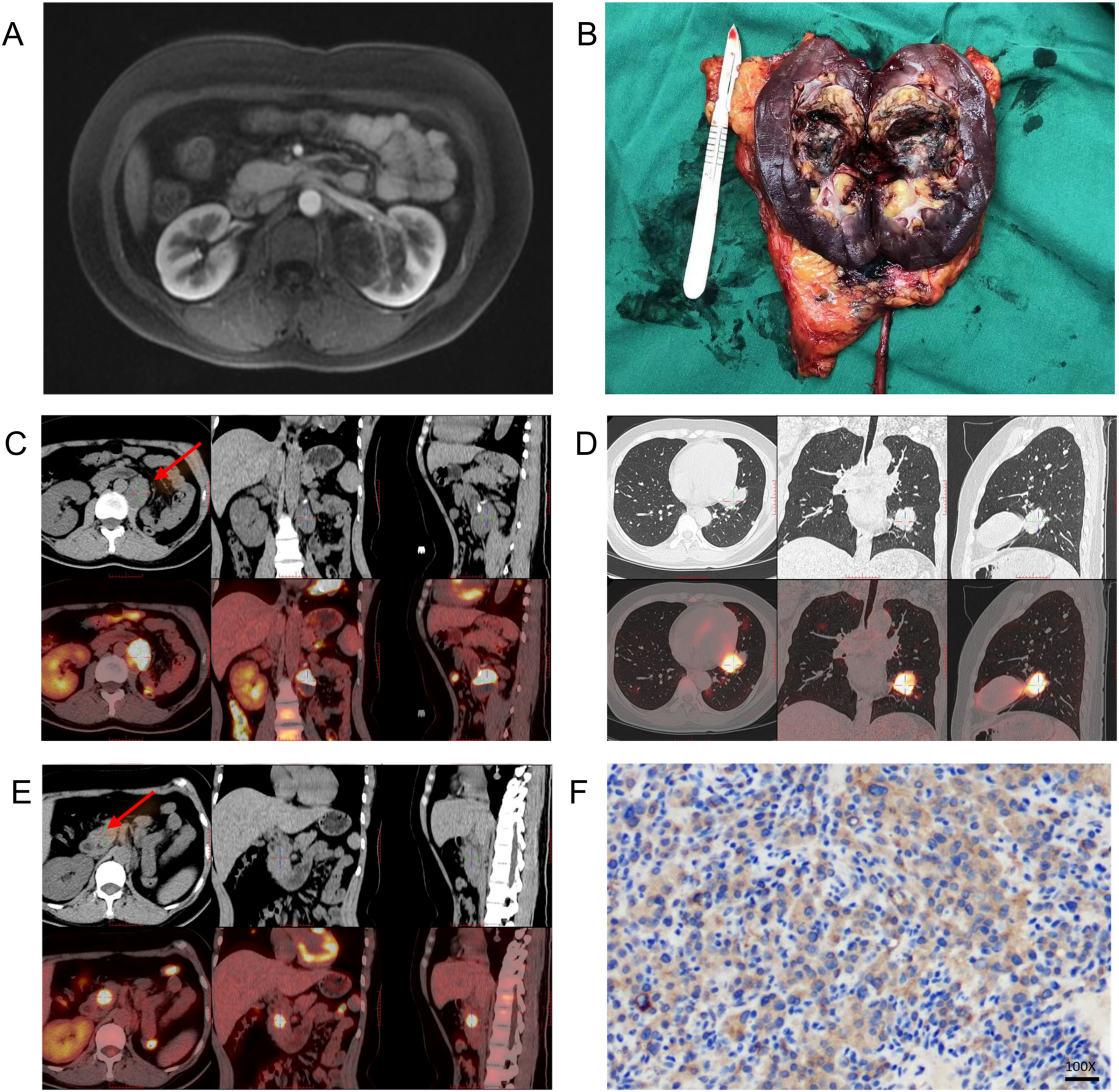
Preoperative and first postoperative follow-up PET-CT scans of the patient. **(A)** Preoperative MRI from 2023; **(B)** Surgical specimen following radical nephrectomy; **(C)** Mass in the left renal surgical field, with indistinct borders at the origin of the left psoas major muscle, showing increased FDG uptake and an SUVmax of approximately 27.1; **(D)** Mass in the anteroinferior basal segment of the left lower lobe, with increased FDG uptake and an SUVmax of approximately 16.7; **(E)** Mass in the pancreatic head, with increased FDG uptake and an SUVmax of approximately 13.5; **(F)** Immunohistochemical examination: PD-L1 negative.

**Figure 2:**
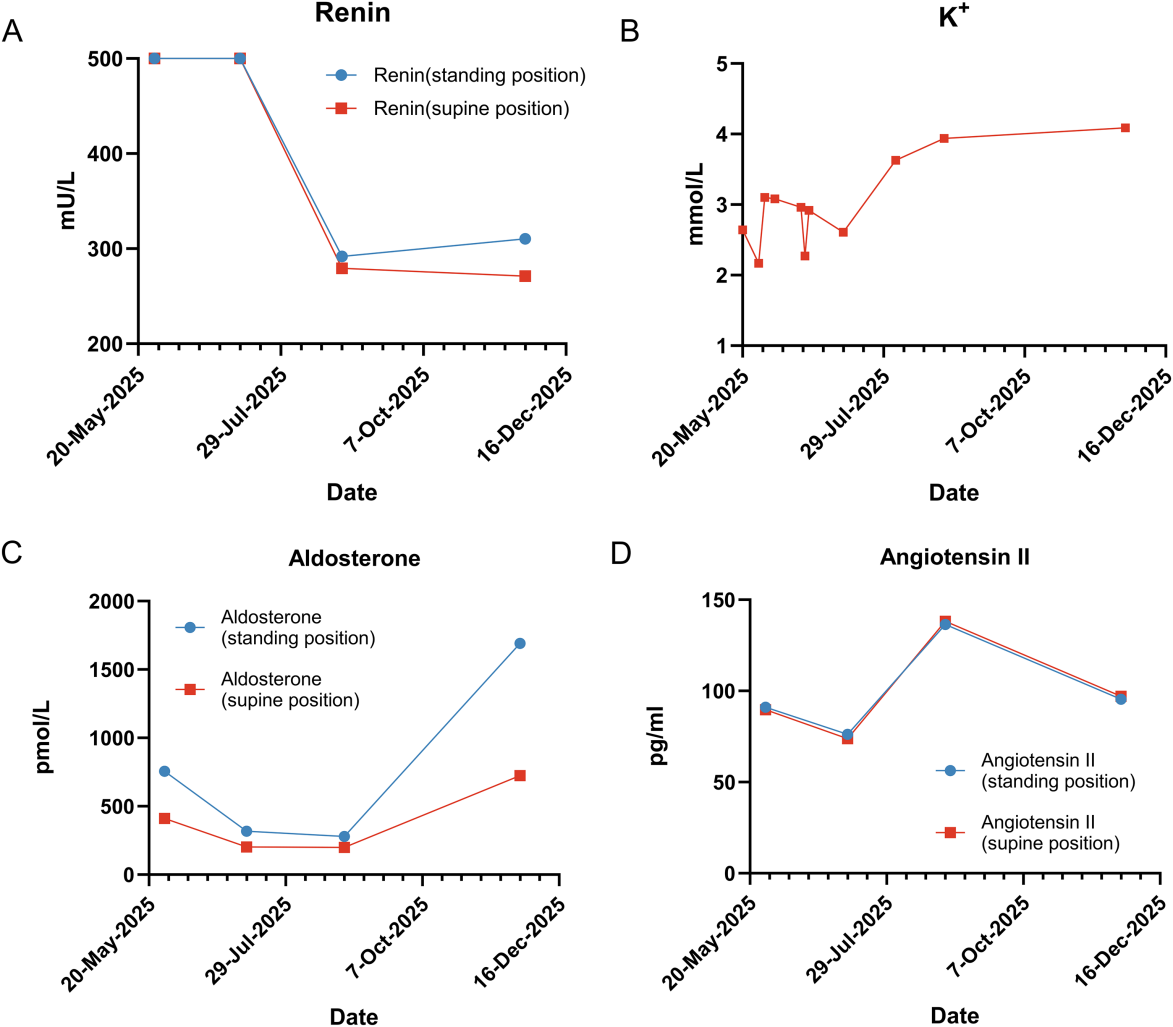
Trend chart of K+ ion and related hormone levels during patient treatment. **(A–D)** Laboratory tests: Renin, K+, Aldosterone, Angiotensin II.

A 32-year-old female presented in November 2023 with sudden left flank pain. Preliminary imaging shows a mass within the left renal pelvis, which was misinterpreted as renal pelvis carcinoma. Although she exhibited severe hypertension (189/122 mmHg) and hypokalemia (2.24 mmol/L), these metabolic cues were not initially prioritized. She underwent a radical left nephrectomy and ureterectomy in December 2023 (**Figures 1A, B**). The patient remained stable for over a year but developed lumbar discomfort in January 2025. Imaging confirmed a recurrent mass in the surgical bed, bilateral pulmonary metastases, and pancreatic head involvement (**Figures 1C–E**). Following a biopsy of the lung lesion and a multidisciplinary review of the original pathology, the diagnosis was revised to malignant JGCT (Reninoma) based on the tumor’s nature and the recurrence of the renin-related triad, **Figure 2** shows the trends in relevant hormone levels and potassium levels as measured during the patient’s treatment.

In June-July 2025, the patient underwent a lung tumor biopsy at the Department of Thoracic Surgery, which revealed a pathological nature consistent with the tumor in the renal region. Consequently, a resection of the lung metastasis was performed, and adjuvant therapy was initiated to control disease progression.

**Figure 3:**
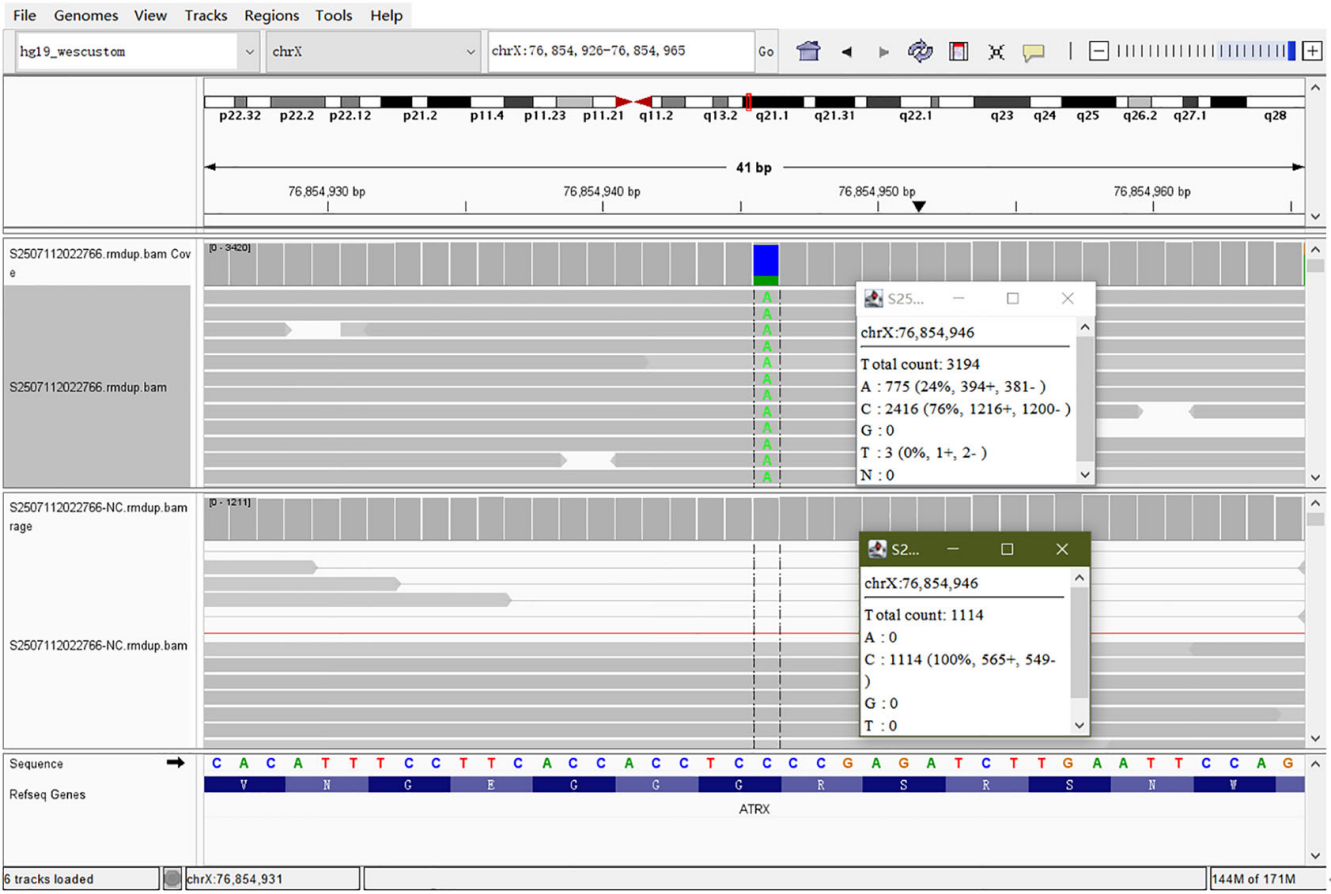
Schematic diagram of patient genetic testing results: IGV visualization of the ATRX (p.G1964*) mutation in the patient’s tumor tissue. The upper panel displays the tumor sample (S2507112022766), showing a G>T substitution (represented as C>A on the displayed strand) at position chrX:76,854,946. The variant allele frequency (VAF) is approximately 24% (775 out of 3194 total counts). This mutation results in a premature stop codon at amino acid position 1964 (p.G1964*). The lower panel shows the matched normal control sample (S2507112022766-NC), where no variant was detected (100% wild-type C/G), Confirm that this mutation is a somatic mutation.

Initial systemic therapy included VMAT radiotherapy, EP chemotherapy (etoposide + cisplatin), and toripalimab immunotherapy. By August 2025, the disease was evaluated as stable (SD). To further optimize the treatment plan, next-generation sequencing (NGS) (**Figure 3**) and related immunohistochemical tests were performed. The analysis results showed:

ATRX mutation: p.G1964* (premature termination), a Class II mutation.

Variant Allele Frequency (VAF) (8): % (Reported result: 23.73%) (**Figure 3**).

PD-L1 Status: Negative via immunohistochemistry (IHC) (**Figure 1F**).

**Figure 4:**
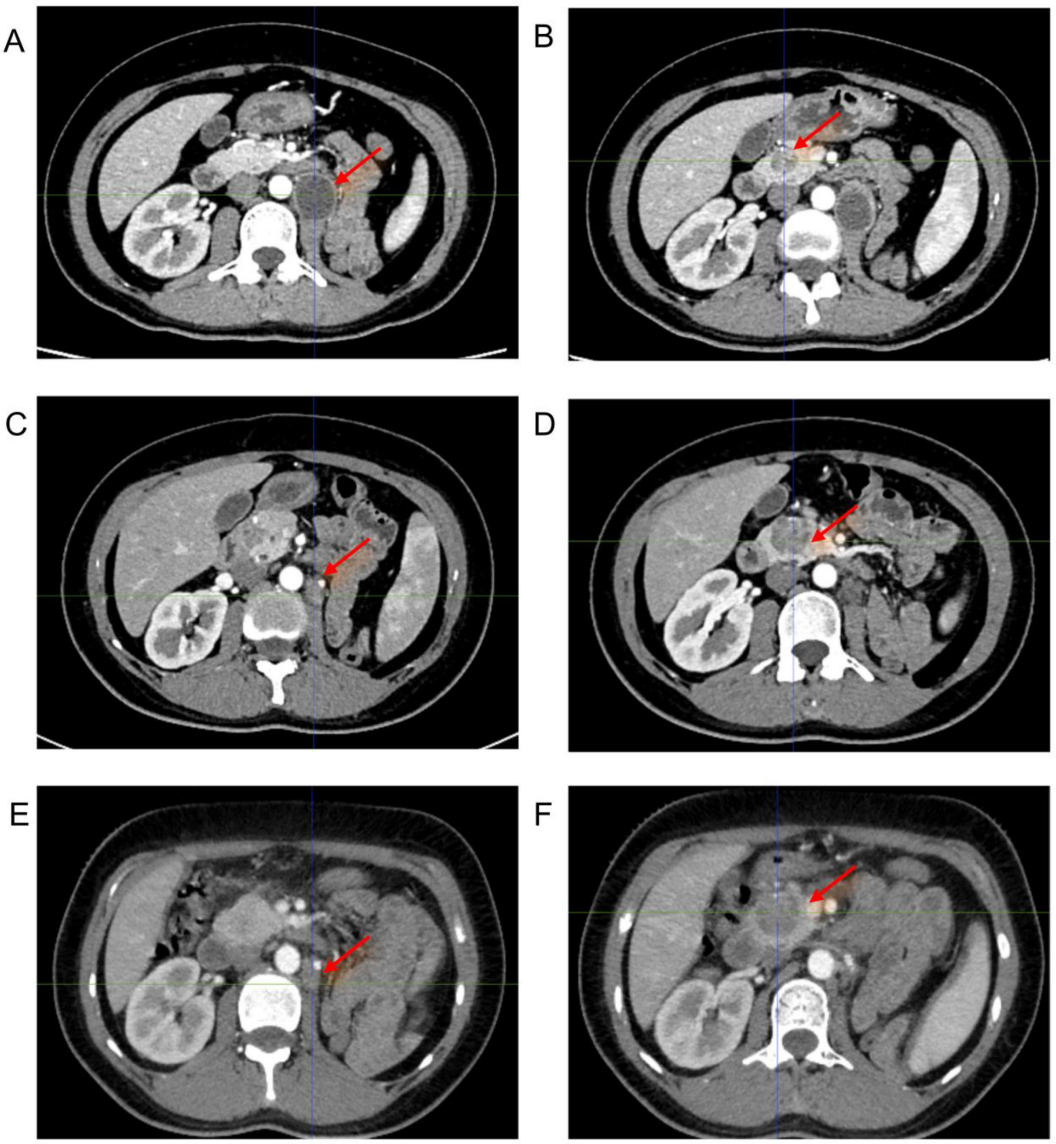
Contrast-enhanced CT before oral olaparib administration and two follow-up contrast-enhanced CT scans after drug therapy. **(A, B)** In August 2025, prior to treatment with olaparib, the tumor in the renal region was large, measuring 3.6 x 2.8 cm, and the tumor in the pancreatic head measured 2.3 cm; **(C, D)** November 2025: The renal tumor measured 2.0 x 1.2 cm, and the pancreatic head tumor measured 2.6 x 2.4 cm; **(E, F)** December 2025: The renal tumor had decreased slightly in size compared to November, and the pancreatic head tumor measured 2.7 x 2.6 cm.

Based on the HRR pathway defect, the patient began targeted therapy with olaparib (300 mg twice daily) in September 2025. A follow-up in November 2025 showed a reduction in the renal region tumor, though the pancreatic lesion increased and pulmonary metastases persisted. December 2025: Follow-up revealed that the recurrent tumor in the renal surgical site had resolved compared to previous findings, while the mass in the pancreatic head region persisted (**Figures 4A–F**), She then underwent one session of radiation therapy and continued treatment with olaparib.

## 3 Clinical and treatment timeline

**Figure 5:**
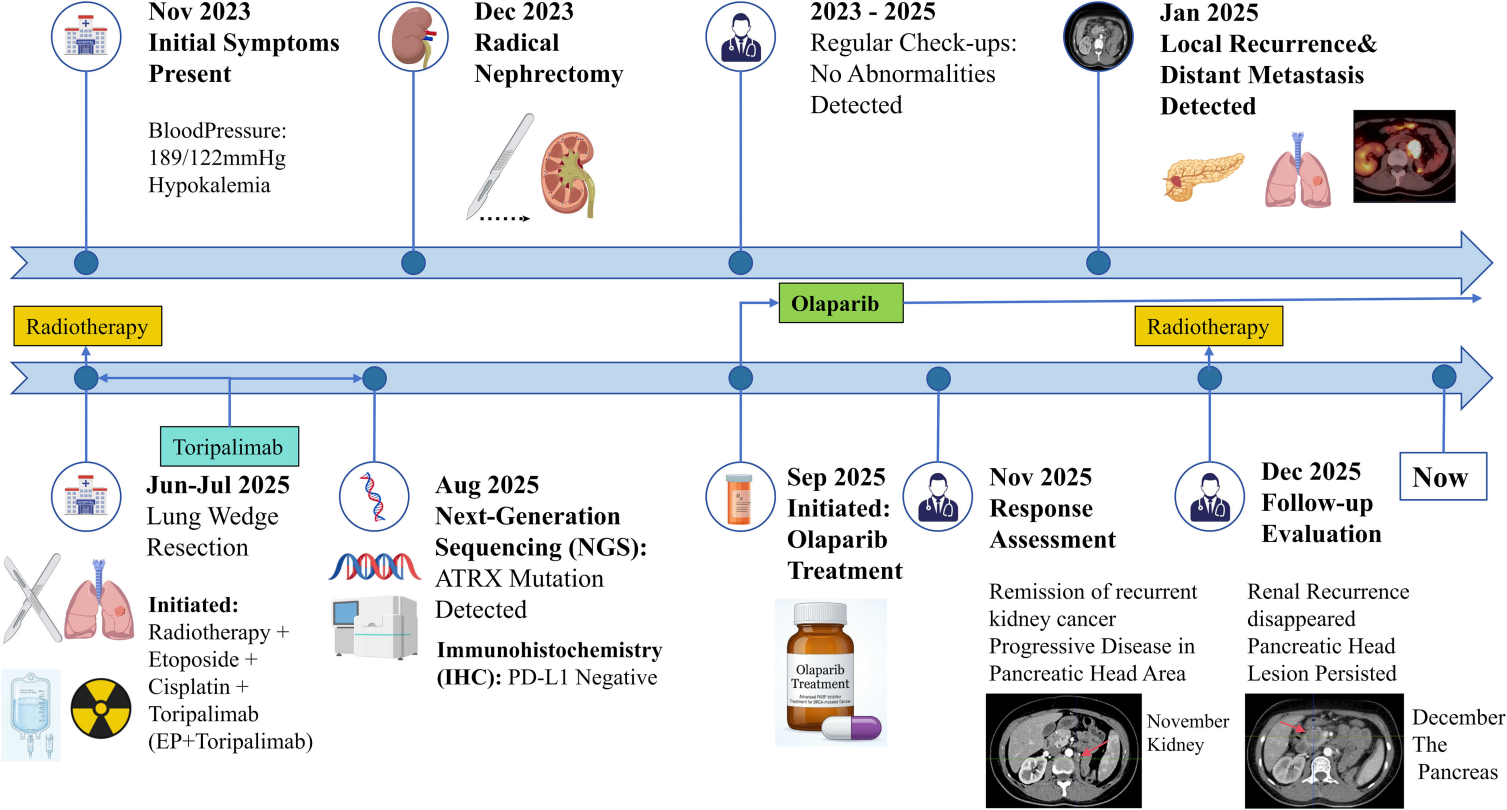
Clinical and treatment timeline.

The chart below shows the patient’s treatment timeline (**Figure 5**).

## 4 Discussion

### 4.1 Diagnostic and pathological considerations

**Figure 6:**
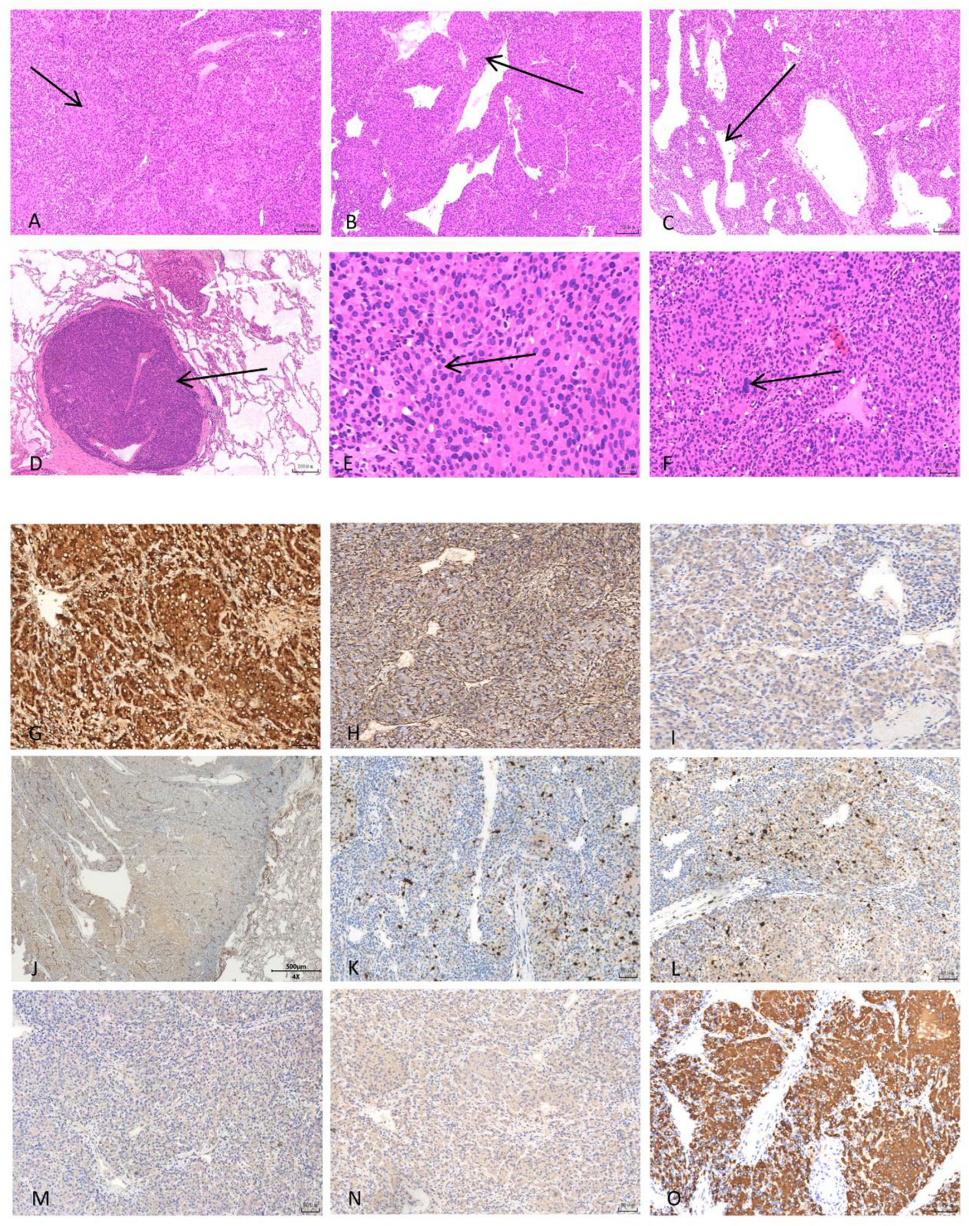
Pathology and immunohistochemistry examination. Pathological Morphological Features (ABC&F: magnification ×100μm, D: magnification×200μm. E: magnification ×50μm): **(A)** The tumor tissue is arranged in solid, patchy patterns; **(B)** The tumor tissue also exhibits a band-like arrangement; **(C)** The tumor tissue exhibits an endothelial-like arrangement; **(D)** Tumor metastasis within lung tissue (black arrow) with hemangiomatous thrombus formation (white arrow); **(E)** Under high magnification, the tumor cells appear round or oval, with dense chromatin and visible pathological mitotic figures; **(F)** Some tumor cells show a perinuclear halo, marked cellular atypia, and visible nucleoli; Immunohistochemical characteristics of malignant reninoma (Figure J, magnification ×500 μm; other figures, magnification ×50 μm): **(G)** Renin shows strong positive expression in the tumor; **(H)** Desmin shows moderate positive expression in the tumor; **(I)** Calponin shows weak positive expression in the tumor; **(J)** CD34 shows moderate positive expression in the tumor (lung tissue); **(K)** p53 shows focal moderate positive expression in the tumor; **(L)** Ki67 showed approximately 40% positive expression in the tumor; **(M)** Vimentin showed weak positive expression in the tumor; **(N)** SMA showed weak positive expression in the tumor; **(O)** Syn showed strong positive expression in the tumor.

Malignant reninoma is frequently misdiagnosed due to its rarity and radiological similarity to other renal carcinomas (9). The patient has other relevant potential positive markers (**Figure 6**), but the diagnosis hinges on recognizing the hyperreninemic hypertension syndrome and specific IHC markers. In this case, the tumor displayed strong cytoplasmic staining for Renin and diffuse positivity for CD34, suggesting characteristics of vascular endothelial or mesenchymal origin, confirming its juxtaglomerular cell origin (4, 10, 11).

### 4.2 Review of treatment strategies

Surgery is usually curative for localized reninoma (5, 12). However, for metastatic disease, treatment is largely experimental. Previous systemic approaches have included platinum-based chemotherapy and various immunotherapies, like immune checkpoint inhibitors and tyrosine kinase inhibitor are also used to treat metastatic clear cell carcinoma (13–15). Previous studies have confirmed the presence of characteristic NOTCH1 gene rearrangements in reninoma (7), Other single-cell RNA sequencing results indicate that the renin gene (REN) and target genes of the Notch signaling pathway (such as HEY1 and HES1) are significantly upregulated in reninoma cells (11), Reports indicate that the disease is characterized by monosomic deletions of chromosomes 9 and 11 (deletion type), accompanied by acquired polyploidy of chromosomes 10, 13, 17, and the X chromosome. Other mutations: In a small number of cases, genetic alterations associated with the MAPK-RAS pathway have been reported (10, 16). However, for this patient, Given the patient’s PD-L1 negative status and limited response to toripalimab, the identification of a targetable ATRX mutation provided a critical shift toward biomarker-guided precision therapy (17). Previous clinical trials have demonstrated the efficacy of olaparib in the maintenance treatment of metastatic pancreatic cancer (18–20); we plan to continue olaparib treatment for this patient (21).

### 4.3 The use of olaparib in cancer treatment

The use of olaparib in this case was predicated on the principle of synthetic lethality. Olaparib inhibits PARP enzymes, which are vital for single-strand break repair. In cells with homologous recombination repair (HRR) defects—such as those harboring ATRX mutations—these unrepaired breaks lead to lethal double-strand breaks during DNA replication (22). While olaparib was first approved for BRCA-mutated cancers (23), recent clinical trials have shown that olaparib exhibits good antitumor activity in patients with metastatic breast cancer and germline BRCA mutations, and metastatic Castration-Resistant Prostate Cancer (8, 24). The final overall survival results showed that olaparib was generally well tolerated, with no evidence of cumulative toxicity during long-term exposure (25). evidence suggests its efficacy extends to other HRD-positive tumors, including those with ATM, CDK12, and ATRX mutations (8, 26, 27).

### 4.4 Differential response and resistance mechanisms

Studies have shown that metastatic lung adenocarcinoma, a type of non-small cell lung cancer, responds well to olaparib (28). There have also been previous studies on the use of PARP inhibitors in the treatment of pancreatic cancer (29). The pathological findings from the biopsies of the patient’s lung and pancreatic tumors both indicate that they are metastases from the recurrent kidney tumor. The differential response—regression in the kidney but growth in the pancreas—highlights the challenge of tumor heterogeneity. Metastatic sites may possess different subclonal architectures or secondary mutations that bypass PARP inhibition. Furthermore, acquired resistance is common in long-term PARP inhibitor use (30). Recent research indicates that sequential combination with ATR inhibitors (e.g., ceralasertib) may overcome this resistance by blocking the replication stress response (31). One study proposed that disrupting DNPH1—a nucleotide salvage factor that eliminates cytotoxic 5-hydroxymethyl-2’-deoxyuridine (hmdU)—increases the sensitivity of HR-deficient and BRCA-deficient cells to PARP inhibitors, thereby offering a new therapeutic strategy for metastatic tumors associated with ATRX mutations (32), offering a potential next step for this patient.

## 5 Conclusion

Malignant reninoma remains a clinical challenge requiring multidisciplinary management. This case underscores the utility of NGS in identifying actionable molecular defects like ATRX mutations in rare cancers. While olaparib provided significant local control, the mixed systemic response emphasizes the need for further research into combination therapies and mechanisms of resistance in HRD-positive non-BRCA tumors.

## Data Availability

The original contributions presented in the study are included in the article/supplementary material. Further inquiries can be directed to the corresponding authors.
